# EBV‐Positive Sinonasal Plasma Cell Neoplasms: A Case Series With Favorable Outcomes in Elderly Males

**DOI:** 10.1002/jha2.70418

**Published:** 2026-10-01

**Authors:** Izel Okcu, Husam A. Jum'ah, Patrick B. Johnston, David J. Inwards, Suzanne R. Hayman, Stephen M. Ansell, Thomas E. Witzig, Thomas M. Habermann, Grzegorz S. Nowakowski, Yucai Wang, Rebecca L. King

**Affiliations:** ^1^ Division of Hematology Mayo Clinic Rochester Minnesota USA; ^2^ Department of Laboratory Medicine and Pathology University of Cincinnati College of Medicine Cincinnati Ohio USA; ^3^ Department of Laboratory Medicine and Pathology Mayo Clinic Rochester Minnesota USA

**Keywords:** Epstein–Barr virus, immunocompetent host, plasma cell neoplasms, plasmablastic lymphoma, plasmacytoma, sinonasal neoplasms

## Abstract

**Introduction:**

Epstein–Barr virus positive plasma cell neoplasms in immunocompetent hosts typically behave like aggressive lymphomas, often classified as plasmablastic lymphoma. We evaluated six sinonasal neoplasms in elderly males with unusually indolent courses at Mayo Clinic.

**Methods:**

A single‐center, retrospective review of plasmablastic lymphoma cases identified six cases with unusually favorable outcomes. We analyzed clinical presentations, pathologic features, and treatment outcomes.

**Results:**

All patients were male with age range 61‐80 years. Five patients had Ann Arbor stage IE disease and one had Stage IV; all lacked B symptoms, and LDH was normal in five. Histologic evaluation revealed mixed mature plasma cell and plasmablastic features in three cases and pure plasmablastic morphology in three. All cases demonstrated EBER and CD138 positivity with monotypic light chain restriction (3 *κ*, 3 *λ*). Four patients recieved systemic chemotherapy, one had local radiation alone, and one was observed. No patients died of this disease within the follow‐up period.

**Conclusions:**

These neoplasms show less aggressive behavior than typical plasmablastic lymphoma, suggesting this sinonasal disease represents a distinct indolent subset of EBV positive PCNs.

**Trial Registration:**

The authors have confirmed clinical trial registration is not needed for this submission.

1

Epstein–Barr virus (EBV)‐positive plasma cell neoplasms are uncommon tumors whose clinicopathologic spectrum is not well described in immunocompetent hosts. Most represent aggressive diseases, with clinical behavior similar to mature aggressive lymphomas rather than plasma cell neoplasms like multiple myeloma (MM). They are classified as lymphomas in current schemes and include entities such as plasmablastic lymphoma (PBL), ALK‐positive large B cell lymphoma, HHV8‐positive large B cell lymphoma, and primary effusion lymphoma [1, 2]. PBL, the most common, classically arises in immunodeficiency (e.g., HIV or posttransplant states) but can present in immunocompetent elderly individuals [3]. The disease follows an aggressive course with a poor prognosis, regardless of HIV status [4].

In contrast to these “lymphomas,” MM and plasmacytomas may rarely be EBV‐positive, although EBV is rarely assessed diagnostically in immunocompetent hosts [5, 6, 7]. Case series of EBV‐positive plasmacytomas in immunocompetent hosts have been reported and show survival similar to EBV‐negative plasmacytoma, rather than the aggressive course expected of PBL [8]. EBV‐positive plasmacytoma is not recognized as a distinct entity in the ICC or WHO fifth edition and its diagnostic criteria remain vague. In published series, most plasmacytomas show mature plasmacytic cytology without marked atypia [6]; however, plasmablastic features are reported in ∼4%–8% and ∼30%–40% of EBV‐positive cases [7, 9].

The Mayo Clinic Institutional Review Board approved this study with an informed consent waiver.We conducted a single‐center retrospective review of the Mayo Clinic institutional database, identifying all cases diagnosed as PBL between 2002 and 2025. Cases were subsequently filtered for primary sinonasal anatomical involvement and evaluated for EBV status via EBER in situ hybridization. Immunocompetency was verified through structured medical record review, confirming negative HIV status, absence of immunosuppressive therapy, and no history of transplantation. These six identified cases represent the complete institutional cohort of EBV‐positive sinonasal plasma cell neoplasms exhibiting an indolent clinical course during the review period. We hypothesize that these cases may be better considered within the spectrum of EBV‐positive plasmacytoma, and compare their clinicopathologic features with criteria for PBL and EBV‐positive plasmacytoma, with implications for diagnosis and management.

The patients' ages ranged from 61 to 80 years. All cases were initially diagnosed as PBL involving the sinonasal tract, including the paranasal sinuses, nasal turbinate, or nasal cavity. Clinical features are summarized in Table 1. Patient 6 had a plasmacytoma treated with radiation 21 years prior.

**TABLE 1:** Summary of clinical features of six cases of EBV‐positive sinonasal neoplasms initially diagnosed as plasmablastic lymphoma.

Patient and disease identifiers						
Age/sex	66/M	74/M	69/M	61/M	80/M	62/M
Presentation site	Paranasal sinuses	L Inferior turbinate	R nostril	R Inf. Turbinate	R nasal cavity	L nasal cavity
Symptom duration prior to diagnosis	3 months	7 months	2 weeks	5 months	6 months	N/A
**Systemic vs. localized disease**						
Ann arbor stage	IE	IE	IV	IE	IE	IE
Bone Marrow Involvement (%)	Negative	Negative	Positive (5%)	Negative	Negative	Negative
Paraprotein (M‐Spike)	N/A	Negative	Negative	Negative	N/A	N/A
Serum free light chains and ratio	N/A	WNL	N/A	N/A	N/A	N/A
**Aggressive vs. indolent features**						
HIV status	Negative	Negative	Negative	Negative	Negative	Negative
ECOG performance status	0	0	0	0	2	1
B symptoms (present/absent)	Absent	Absent	Absent	Absent	Absent	Absent
LDH level (U/L)	WNL	WNL	WNL	WNL	WNL	Elevated
Cytopenia	Absent	Absent	Absent	Absent	Absent	Absent
**Treatment and outcome**						
Treatment administered	CHOP x6, IT MTX, XRT	R‐CHOP, IT MTX, RT	Expectant Management	DA‐EPOCH, RT	RT (Refractory to R/Valtrex)	CHOEP, Pembrolizumab, RT
Follow‐up duration	5 years	10 years	13 years	9 years	6 months	22 months
Status at last follow‐up	Died	Alive, NED	Alive, NED	Alive, NED	Alive	Died

*Note*: Ann Arbor stage, lymphoma staging system (stages I–IV; “E” denotes extranodal involvement). Patient 6: Subsequent therapies (e.g., additional pembrolizumab) were administered for a concurrent cutaneous T‐cell lymphoma rather than sinonasal disease.

Abbreviations: B symptoms, systemic lymphoma‐related symptoms (fever, night sweats, weight loss); ECOG, Eastern Cooperative Oncology Group performance status (0 = fully active, 5 = death); HIV, human immunodeficiency virus; Inf., inferior; LDH, lactate dehydrogenase; M, male; M‐spike, monoclonal protein spike detected on serum protein electrophoresis; NED, no evidence of disease; R/L, right/left; WNL, within normal limits.

Five patients had Ann Arbor stage IE disease and one (Patient 3) had Stage IV; all lacked B symptoms, and LDH was normal in five.

First‐line treatments administered were as follows: Patient 1 received CHOP, intrathecal methotrexate (IT MTX), and radiation therapy (RT); Patient 2, R‐CHOP, IT MTX, and RT; Patient 4 received dose‐adjusted EPOCH and RT; Patient 5, rituximab and valacyclovir (refractory) followed by RT; and Patient 6, CHOEP, pembrolizumab, and RT. Following an excisional biopsy of the nostril mass and a staging bone marrow assessment, Patient 3 was managed with active surveillance alone. All therapies were first‐line PBL management; none received salvage therapy.

The follow‐up duration for the cohort ranged from 6 months to 13 years. At the last follow‐up, four of the six patients were alive. Patient 1 died 5 years after diagnosis from esophageal cancer; PBL was in remission at death. Patient 6 achieved complete remission from PBL and died ∼22 months later from cutaneous T‐cell lymphoma. The status of the surviving patients was as follows: alive with no evidence of disease (NED) at 10 years (Patient 2), 13 years (Patient 3), and 9 years (Patient 4); and alive at 6 months with disease status not yet assessed (Patient 5).

Slides were available for retrospective review in four cases and only reports were reviewed for the other two. The pathologic findings across the six cases shared core immunophenotypic features while demonstrating morphologic heterogeneity. Three cases (Patients 1, 2, and 3) showed areas of mature plasma cells with atypia, and areas of plasmablastic morphology with high N:C and prominent nucleoli (all three cases re‐reviewed; Figure 1A,B). The remaining three cases were purely plasmablastic (one by re‐review, two by pathology report description; Figure 1C). High‐grade lymphoma features were inconsistent; a “starry sky” pattern was identified only in Patient 1 (Figure 1D), and necrosis was present only in Patient 3 (Figure 1B). The Ki‐67 proliferation index, assessed in only one case (Patient 4), was 50%.

**FIGURE 1:**
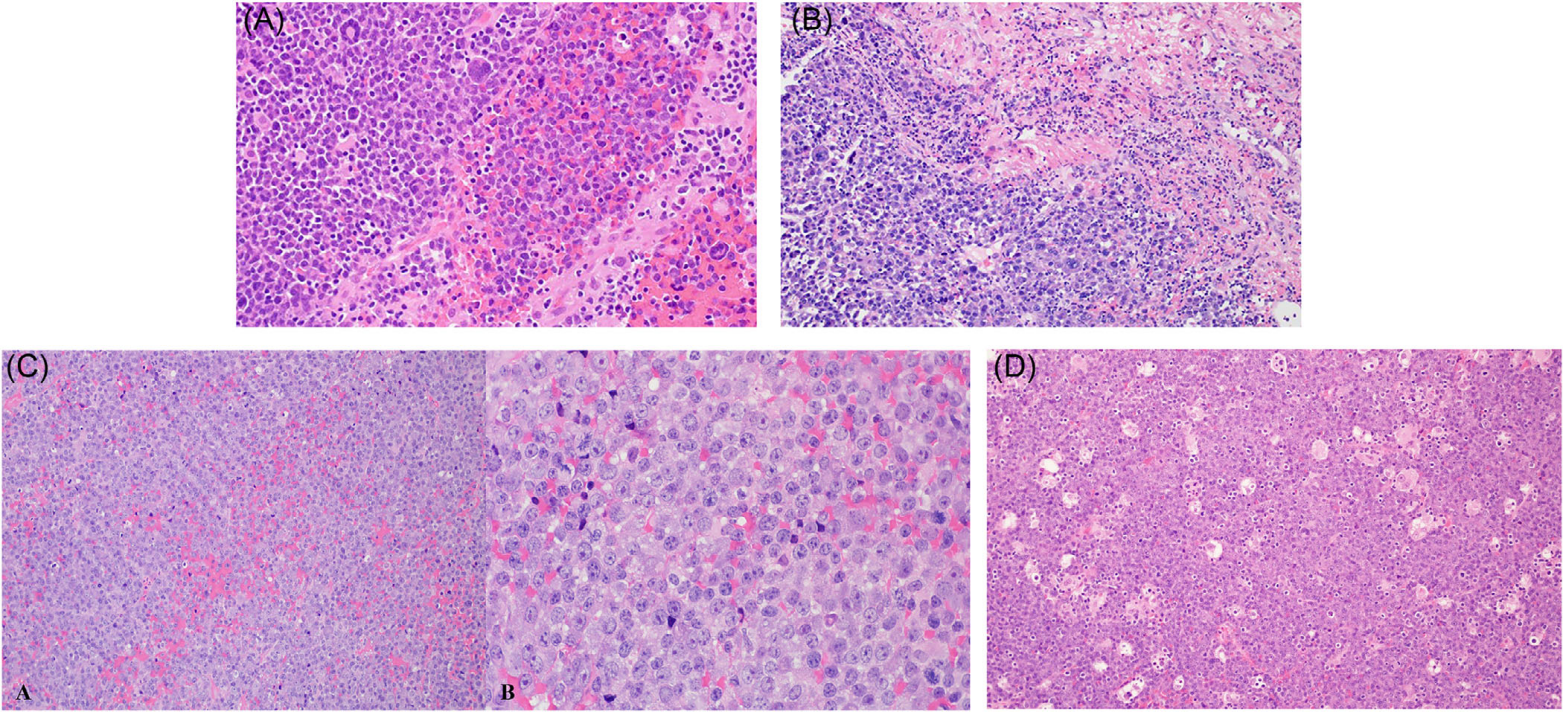
Histopathologic spectrum of sinonasal EBV‐positive plasma cell neoplasms. (A) Case 1: Plasmablastic cells, with areas of mature appearing plasma cells. Some of the cells show prominent nucleoli, and binucleate and multinucleate forms are also present. (H+++E; 400 ×). (B) Case 3: Plasmablastic cells, with areas of mature appearing plasma cells and necrosis. (H+++E; 200 ×). (C) Case 6: Sheets of plasmablastic cells with large eccentrically placed round nuclei, vesicular chromatin, prominent nucleoli, and modest amount of eosinophilic cytoplasm with prominent mitotic figures (Panel A: H+++E; 200 ×; Panel B: H+++E; 600 ×). (D) Case 1: Starry sky appearance (H+++E; 200 ×).

Immunophenotypically, all six cases were positive for EBV (EBER) and the plasma cell marker CD138. All cases also demonstrated monotypic light chain expression, with three cases showing *κ* restriction and three showing *λ* restriction. Expression of B‐cell markers varied; CD20 was negative in four cases with patchy expression in one, while PAX5 was negative in the two cases in which it was assessed. Additional details are summarized in Table S1.

FISH and molecular studies were not performed in sinonasal samples, although one patient (Patient 3) had a *t*(11;14) identified by FISH in the bone marrow.

Our series of six HIV‐negative elderly males with EBV‐positive sinonasal neoplasms, initially diagnosed as PBL, suggests that these cases exhibit less aggressive behavior than typical PBL. This finding suggests some may be more appropriately classified as EBV‐positive plasmacytoma and highlights the lack of definitive histopathologic criteria for making this distinction. Our data expand upon rare reports suggesting that while EBV‐positive plasma cell neoplasms in younger patients or at extranasal sites typically present as aggressive PBL, the sinonasal tract in elderly males may host a biologically distinct, more indolent subset [6, 8, 10]. Notably, the majority of patients had excellent long‐term outcomes, including Patient 2 (10 years NED), Patient 3 (13 years in clinical remission despite initial marrow involvement), Patient 4 (9 years NED), and Patient 6 (2 years NED following treatment for a second sinonasal lesion and 22 years post‐initial plasmacytoma). This favorable long‐term clinical course contrasts sharply with the generally aggressive nature and poor prognosis typical of PBL as initially characterized [3] and further defined [11]. Even Patient 1, who died at 5 years, exceeded typical PBL survival expectations based on some historical data.

Pathologically, these cases underscore the diagnostic challenge due to overlapping features between EBV‐positive plasmacytoma with anaplastic/plasmablastic changes and PBL demonstrating plasmacytic differentiation [6, 8, 12]. Zhou et al. [8] proposed criteria to aid in this distinction, suggesting a PBL diagnosis is favored if two or more of the following features are present: a “starry‐sky” pattern, coagulative tumor necrosis, a Ki‐67 proliferation index greater than 75%, and absence of light chain expression. Conversely, absence of ≥ 3 features favors EBV‐positive plasmacytoma. Applying these proposed criteria to our series reveals a variable fit, hampered by a lack of available material for review in all cases. A “starry‐sky” pattern was present only in Patient 1, who had an intermediate outcome. This feature was absent in Patients 2 and 3 (who had excellent outcomes), and in Patient 6, and was not evaluated in the remaining cases. Tumor associated necrosis was noted in Patient 3, who nonetheless had an excellent long‐term outcome; it was absent in Patients 1, 2, and 6, and not evaluated in Patients 4 and 5. A Ki‐67 proliferation index was not studied in the majority of our cases; it was only evaluated in Patient 4, which had a Ki‐67 of 50%. Notably, all cases in our series demonstrated light chain expression, noted in the Zhou series to be more common in plasmacytoma than PBL. Thus, most cases did not meet multiple criteria favoring PBL, though data were incomplete.

The presence of mature‐appearing plasma cells or variably sized plasmacytoid cells alongside plasmablastic forms (observed in Patients 1, 2, and 3) is compatible with the known morphological spectrum of EBV‐positive plasmacytoma [7, 8]. The LMP1 positivity in a subset of cells in Patient 4 is noteworthy, as that Zhou et al. [8] reported LMP1 negativity in their EBV‐positive plasmacytoma cohort, although conclusions cannot be drawn from only one case. While *MYC* rearrangements are characteristic of many PBL cases [13], they can also be seen in MM/plasmacytoma and this was not assessed in any of our cases.

Limitations include retrospective design, lack of baseline SPEP/sFLC data, and incomplete pathology material for re‐review and molecular profiling. Despite this, our extensive follow up on these patients and their uniform clinical presentation suggest that these cases may represent a clinically indolent subset of EBV‐positive plasma cell neoplasms in spite of their aggressive pathologic appearance. This interpretation is supported by the presence of *t*(11;14) in a clonal bone marrow plasma cell population in one patient, and by the history of a prior plasmacytoma in Patient 6 preceding a lesion diagnosed as PBL. The favorable outcomes seen in our cohort, achieved with standard lymphoma regimens or even expectantly, imply that these neoplasms are either an unusually responsive subset of PBL in this specific demographic or, more likely, a biologically distinct, less aggressive EBV‐driven plasma cell neoplasm. Recognizing this particular clinicopathological profile may guide therapeutic decisions, potentially averting unnecessary treatment intensification/escalation. Comprehensive molecular analyses, as advocated by Zhou et al. [8], remain crucial for future studies to better delineate these EBV‐positive sinonasal plasma cell neoplasms and clarify their precise relationship to classical plasmacytoma and PBL.

In conclusion, this case series demonstrates a potentially distinct presentation of EBV‐positive plasma cell neoplasms and suggests the integration of all clinicopathological data for accurate diagnosis and management.

## Author Contributions

Conceptualization: I.O., G.S.N., Y.W., and R.L.K. Methodology: I.O., H.A.J., G.S.N., Y.W., and R.L.K. Data curation: I.O. and H.A.J. Formal analysis: I.O. Writing – original draft: I.O. Investigation: H.A.J. and R.L.K. Writing – review and editing: H.A.J., G.S.N., Y.W., R.L.K., P.B.J., D.J.I., S.R.H., S.M.A., T.E.W., and T.M.H. Validation: G.S.N., Y.W., R.L.K., P.B.J., D.J.I., S.R.H., S.M.A., T.E.W., and T.M.H. Resources: G.S.N., Y.W., R.L.K., P.B.J., D.J.I., S.R.H., S.M.A., T.E.W., and T.M.H. Supervision: R.L.K. All authors approved the final version of the manuscript.

## Funding

The authors have nothing to report.

## Conflicts of Interest

The authors declare no conflicts of interest.

## Supporting information




**Supporting File 1**: jha270418‐sup‐0001‐Table S1.docx

## Data Availability

The data supporting the findings of this study are available from the corresponding author upon request.
