# Supplementary material for: EBV‐Positive Sinonasal Plasma Cell Neoplasms: A Case Series With Favorable Outcomes in Elderly Males
Source: EJHaem. 2026 Oct 1;7(5):e70418. doi: 10.1002/jha2.70418 (PMC13628157; doi:10.1002/jha2.70418)
Supplement: Supplementary file 1 [file JHA2-7-e70418-s001.docx]

Supplementary Table S1: Summary of Pathological Findings of Six Cases of EBV-Positive Sinonasal Neoplasms Initially Diagnosed as Plasmablastic Lymphoma

| **Feature** | **Patient 1** | **Patient 2** | **Patient 3** | **Patient 4** | **Patient 5** | **Patient 6 (Recurrence)** |
| --- | --- | --- | --- | --- | --- | --- |
| **Initial Diagnosis** | PBL | PBL | PBL | PBL | PBL | PBL |
| **Histopathology** |  |  |  |  |  |  |
| Morphology | Mature & Plasmablastic | Mature & Plasmablastic | Mature & Plasmablastic | Plasmablastic | Plasmablastic | Plasmablastic |
| "Starry Sky" Pattern | Yes | No | No | N/A | N/A | No |
| Necrosis | No | No | Yes | N/A | N/A | No |
| Ki-67 Index | NA | NA | NA | 50% | NA | NA |
| **Immunophenotype** |  |  |  |  |  |  |
| EBV (EBER) | Positive | Positive | Positive | Positive | Positive | Positive (Diffuse) |
| LMP1 | NA | NA | NA | Subset Positive | NA | NA |
| Light Chain Restriction | Kappa | Kappa | Lambda | Lambda | Lambda | Kappa |
| CD138 | Positive | Positive | Positive | Positive | Positive | Positive |
| MUM1 | Positive | NA | NA | NA | Positive | NA |
| CD20 | Negative | Positive (patchy) | NA | Negative | Negative | Negative |
| PAX5 | NA | NA | NA | Negative | NA | Negative |
| CD79a | Positive | NA | NA | NA | NA | Positive |
| CD45 | Positive (dim) | Positive (dim) | NA | NA | NA | Negative |
| CD19 | Negative | NA | NA | NA | NA | Negative |
| CD56 | NA | NA | NA | NA | NA | Positive (wk/part) |
| HHV8 | NA | NA | NA | NA | NA | Negative |
| Other IHC | CD138+, CD79a+, MUM1+ | CD138+, CD45(wk)+, CD20(patchy)+ | CD138+ | CD138+, LMP1(subset)+, CD20- | CD138+, MUM1+, CD20- | CD138+, CD79a+, CD56(wk/part)+ |
| Abbreviations: EBV, Epstein–Barr virus; EBER, Epstein–Barr virus–encoded RNA; Ki-67, proliferation index; MUM1, multiple myeloma oncogene 1; PAX5, paired box gene 5; CD, cluster of differentiation; N/A, not available; wk/part, weak/partial; PBL, plasmablastic lymphoma; LMP1, latent membrane protein 1; IHC, immunohistochemistry; HHV8, human herpesvirus 8; CD138, CD79a, CD20, CD19, and CD45, cluster of differentiation surface markers used for immunophenotypic characterization. | | | | | | |
